# Comparison of the accuracy of methods of computational haplotype inference using a large empirical dataset

**DOI:** 10.1186/1471-2156-5-22

**Published:** 2004-08-03

**Authors:** Ronald M Adkins

**Affiliations:** 1 Children's Foundation Research Center and Center of Genomics and Bioinformatics, University of Tennessee, Memphis, TN, USA

## Abstract

**Background:**

Analyses of genetic data at the level of haplotypes provide increased accuracy and power to infer genotype-phenotype correlations and evolutionary history of a locus. However, empirical determination of haplotypes is expensive and laborious. Therefore, several methods of inferring haplotypes from unphased genotypic data have been proposed, but it is unclear how accurate each of the methods is or which methods are superior. The accuracy of some of the leading methods of computational haplotype inference (PL-EM, Phase, SNPHAP, Haplotyper) are compared using a large set of 308 empirically determined haplotypes based on 15 SNPs, among which 36 haplotypes were observed to occur. This study presents several advantages over many previous comparisons of haplotype inference methods: a large number of subjects are included, the number of known haplotypes is much smaller than the number of chromosomes surveyed, a range in values of linkage disequilibrium, presence of rare SNP alleles, and considerable dispersion in the frequencies of haplotypes.

**Results:**

In contrast to some previous comparisons of haplotype inference methods, there was very little difference in the accuracy of the various methods in terms of either assignment of haplotypes to individuals or estimation of haplotype frequencies. Although none of the methods inferred all of the known haplotypes, the assignment of haplotypes to subjects was about 90% correct for individuals heterozygous for up to three SNPs and was about 80% correct for up to five heterozygous sites. All of the methods identified every haplotype with a frequency above 1%, and none assigned a frequency above 1% to an incorrect haplotype.

**Conclusions:**

All of the methods of haplotype inference have high accuracy and one can have confidence in inferences made by any one of the methods. The ability to identify even rare (≥ 1%) haplotypes is reassuring for efforts to identify haplotypes that contribute to disease in a significant proportion of a population. Assignment of haplotypes is relatively accurate among subjects heterozygous for up to 5 sites, and this might be the largest number of SNPs for which one should define haplotype blocks or have confidence in haplotype assignments.

## Background

Very rapid and inexpensive methods exist for determining the genotype of diploid organisms at single nucleotide polymorphisms (SNPs). Unfortunately, these high-throughput methods do not provide direct information on which SNP alleles at multiple sites coexist on the same chromosome. Instead, computational methods must be employed to infer the set of SNP alleles that are cosegregating on a single chromosome, referred to as haplotypes. However, the inference of haplotypes from phase-unknown data is computationally difficult, partly due to the fact that the number of possible haplotypes roughly increases as a power of 2 with each additional SNP.

Interest in the accurate inference of haplotype structure from unphased genotypic data has increased tremendously in recent years for several reasons. Relative to analysis of single polymorphisms, haplotypes can greatly improve one's ability to infer the evolutionary history of a DNA region [1,2]. Additionally, haplotypes can provide significant increases in statistical power to detect associations between a phenotype and genetic variation [3-5]. Indeed, several disease associations with haplotypes have been detected that were not apparent from single-site analyses [6-9].

There are three principal computational approaches to inferring haplotypes from unphased SNP data. The most commonly used approach is implementation of the expectation-maximization (EM) algorithm [10]. This method is computationally intensive and is usually combined with various strategies to simplify the task (i.e., by considering only subsets of the sites at a time) or to minimize the number of potential haplotypes that must be considered [11,12]. A more recent alternative is application of Bayesian methods that incorporate prior expectations based upon population genetic principles [13-15]. A third method based on parsimony ("subtraction method"; [16]) has the limitation that haplotypes are assigned only in unambiguous cases [17], and the level of ambiguity generally increases with the number of sites considered or the number of sites at which an individual is heterozygous. This limitation is expected to be significant in large-scale analyses of SNP variation, and for this reason the subtraction method is not considered here. Unfortunately, it is unclear how accurate the EM and Bayesian approaches are or whether the EM or Bayesian method is superior in inferring haplotypes, particularly when applied to empirical data. Data simulation [18] can explore the effect of a wide range of parameters and population dynamics (i.e., linkage disequilibrium, selection, population substructuring) but is unlikely to achieve fully the complex combinations of these effects inherent in empirical data. On the other hand, comparisons using empirical data have been based on as few as six SNPs [17,19] or have employed data sets in which the number of SNPs or known haplotypes equals or greatly exceeds the number of individuals sampled [13,15]. Neither of these situations is likely to be an accurate reflection of the sample sizes or numbers of SNPs that will be assayed with the high-throughput methods available today. To understand the relative performance of the various methods of haplotype inference, there is a need for comparisons that include both larger numbers of polymorphic sites and biologically more complex correlations among the sites. In this study the performance of several leading methods of haplotype inference are compared for a large data set (154 individuals, 15 SNPs) undergoing a combination of mutation, recombination, and gene conversion.

The accuracy of computational haplotype inference improves as the magnitude of linkage disequilibrium (LD) among sites increases [17]. Gene conversion, operating in conjunction with normal recombination, can complicate the normal decay of linkage disequilibrium with distance in a genomic region and can be expected to complicate the computational inference of haplotype structure. This issue has particular relevance to the human growth hormone locus. The five genes of the human growth hormone locus reside within about 45 kb on chromosome 17 [20]. Pituitary growth hormone (GH1) is by far the most thoroughly studied of the genes and lies at the 5' end of the cluster. The remaining four genes, placental growth hormone (GH2) and three chorionic somatomammotropins (CS1, CS2, and pseudogene CS5 or CSHP1), are expressed only from the placenta. The promoter region of GH1 is unusually polymorphic, with 16 SNPs having been identified in a span of 535 bp [21-23]. Most of these SNPs occur at the comparatively small number of sites that exhibit sequence differences among the five genes of the GH locus, and this has been interpreted as evidence of gene conversion [21,23,24]. A survey of 25 SNPs in the entire promoter and coding region of GH1 (Adkins et al. in review) indicates that this bias towards polymorphism at sites of intergenic divergence is quite extreme and supports the hypothesis that gene conversion plays a role in the pattern of variation in the GH1 gene in addition to mutation and recombination. In 154 recruits to the British army, Horan et al. [23] used cloning and sequencing to empirically determine 36 haplotypes based on 15 of the promoter SNPs previously identified (one site identified by [21] was invariant). This study takes advantage of the exhaustive work of Horan et al. [23] to compare the relative accuracy of some of the major implementations of the EM and Bayesian approaches to haplotype inference.

## Results and Discussion

### Characteristics of the data set

The 15 sites studied by Horan et al. [23] span 535 nucleotides in the promoter of GH1, with minor allele frequencies ranging from 0.3–41.2%. Six of the sites can be considered "rare" variants with minor allele frequencies below 5% (0.3–3.6%). Standardized linkage disequilibrium (D'; [25]) among the remaining nine sites ranges from complete linkage disequilibrium (sites -301 and -308; Table 1) to effective linkage equilibrium (i.e., sites -1 and +59). Fallin and Schork [18] identified several characteristics of an unphased set of genotypes that improve the accuracy of haplotype inference, most of which are exhibited by this data set and discussed below. Therefore, this data set probably represents one that is favourable to the accurate inference of haplotypes.

**Table 1:** Linkage disequilibrium (D') among loci with minor allele frequencies ≥ 5%^1^

	Site						
	
Site	-278	-75	-57	-31	-6	-1	+59
-308	1.000	0.653	0.892	0.741	0.458	0.192	0.646
-278		0.857	0.845	0.696	0.820	0.666	0.334
-75			1.000	0.358	0.708	0.561	0.172
-57				1.000	0.872	1.000	1.000
-31					0.410	1.000	0.538
-6						1.000	0.194
-1							0.002

^1 ^Numbering relative to the start of transcription for GH1

Increasing sample size improves the accuracy of inferred haplotypes. In Horan's study [23] 308 chromosomes were surveyed to yield 36 haplotypes, a ratio of 8.6. Three haplotypes can be unambiguously inferred from the 27 fully homozygous individuals, and 11 subjects are heterozygous at only one site, from which an additional 11 haplotypes can be unambiguously inferred. This leaves 116 individuals (232 chromosomes) heterozygous at ≥ 2 sites upon which to attempt to infer the remaining 22 true haplotypes.

The distribution of haplotype frequencies also influences the accuracy of haplotype inference in two ways. First, the presence of some haplotypes at comparatively high frequency increases the chances that those haplotypes can be unambiguously inferred from homozygotes, allowing the alternative haplotype to be inferred with high confidence in compound heterozygotes. Second, the presence of some haplotypes at near-zero frequency allows truly nonexistent haplotypes to be accurately estimated as having zero frequency. The empirical haplotype frequencies in this study exhibit considerable dispersion. Two haplotypes are relatively common (33% and 16%; Table 2). Thirty-one haplotypes have frequencies below 5%, and 19 have frequencies ≤ 1%. In multiple regression analysis, Fallin and Schork [18] found dispersion of haplotype frequencies to be the strongest predictor of the accuracy of haplotype inference.

**Table 2:** Inferred frequencies of haplotypes.

	SNP															Haplotype Frequency					
		
	-476	-339	-308	-301	-278	-168	-75	-57	-31	-6	-1	3	16	25	59	Empirical	Phase, no LD	Phase, with LD	Haplotyper	PL-EM	SNPHAP
	Empirical Haplotypes																				
1	G	G	G	G	G	T	A	T	G	A	A	G	A	A	T	0.334	0.312	0.321	0.325	0.333	0.326
2	G	G	G	G	T	T	A	G	G	G	A	G	A	A	T	0.162	0.166	0.162	0.166	0.181	0.171
3	G	G	T	T	G	T	A	G	G	A	A	G	A	A	T	0.091	0.097	0.097	0.101	0.098	0.102
4	G	G	T	T	G	T	A	G	-	A	A	G	A	A	T	0.052	0.055	0.055	0.049	0.047	0.050
5	G	G	G	G	T	T	G	G	G	G	A	G	A	A	T	0.042	0.052	0.052	0.052	0.049	0.050
6	G	G	T	T	G	T	A	G	-	A	A	G	A	A	G	0.029	0.032	0.032	0.032	0.030	0.030
7	G	G	G	G	T	T	A	G	G	G	T	G	A	A	T	0.026	0.032	0.032	0.032	0.028	0.029
8	G	G	T	T	G	T	A	G	G	G	A	G	A	A	T	0.019	0.016	0.016	0.013	0.016	0.018
9	G	G	G	G	T	T	A	T	G	G	A	G	A	A	T	0.019	0.013	0.013	0.013	0.011	0.011
10	G	G	T	T	G	T	A	G	-	G	A	G	A	A	T	0.019	0.023	0.026	0.023	0.025	0.025
11	G	G	G	G	T	T	G	G	G	G	A	G	G	C	T	0.016	0.016	0.016	0.016	0.014	0.014
12	G	G	G	G	T	T	A	G	G	A	A	G	A	A	T	0.016	0.010	0.006	0.006	0.008	0.008
13	G	-	G	G	T	T	G	G	G	G	A	G	A	A	T	0.016	0.016	0.016	0.013	0.010	0.013
14	G	G	G	G	T	C	A	G	G	G	T	G	A	A	T	0.016	0.016	0.016	0.016	0.016	0.016
15	G	G	T	T	G	T	A	G	G	G	T	G	A	A	T	0.013	0.010	0.010	0.010	0.006	0.009
16	G	G	G	G	T	T	G	G	G	A	A	G	A	A	T	0.013	0.013	0.013	0.016	0.008	0.008
17	G	-	G	G	T	T	A	G	G	G	A	G	A	A	T	0.013	0.013	0.013	0.013	0.011	0.011
18	G	G	G	G	T	T	A	G	-	G	A	G	A	A	T	0.010	-	0.006	0.010	0.007	0.008
19	A	G	G	G	T	T	A	G	G	G	A	G	A	A	T	0.010	0.013	0.013	0.010	0.005	0.010
20	G	G	G	G	G	T	A	G	-	A	A	G	A	A	T	0.010	-	0.003	0.010	0.006	0.005
21	G	G	G	G	T	T	G	G	G	G	A	G	A	A	G	0.010	0.010	0.010	0.010	0.011	0.011
22	G	G	T	T	G	T	A	T	G	A	A	G	A	A	T	0.010	0.013	0.010	0.013	0.007	0.007
23	G	G	G	G	G	T	A	G	G	A	A	G	A	A	T	0.006	0.016	0.013	0.006	0.006	0.008
24	G	G	T	T	G	T	G	G	-	A	A	G	A	A	T	0.006	-	-	-	-	-
25	G	G	T	T	G	T	A	G	G	A	A	G	A	A	G	0.003	-	-	-	0.004	0.004
26	G	G	G	G	T	T	G	G	G	G	T	G	A	A	T	0.003	0.006	0.006	0.006	0.007	0.007
27	G	G	G	G	T	T	A	T	G	A	A	G	A	A	T	0.003	0.003	0.003	-	-	-
28	G	G	G	G	T	T	A	G	-	A	A	G	A	A	T	0.003	-	-	-	-	-
29	A	G	G	G	T	T	A	G	G	A	A	G	A	A	T	0.003	-	-	-	-	-
30	G	-	G	G	T	T	A	G	G	A	A	G	A	A	T	0.003	0.003	0.003	0.003	0.003	0.003
31	G	G	G	G	T	T	G	G	-	G	A	G	A	A	T	0.003	0.010	-	-	-	-
32	G	G	T	T	G	T	G	G	G	G	A	G	A	A	G	0.003	-	-	-	0.002	-
33	G	G	G	G	T	T	A	G	G	G	A	G	G	C	T	0.003	0.003	0.003	0.003	0.004	0.004
34	G	-	G	G	T	C	A	G	G	G	T	G	A	A	T	0.003	0.003	0.003	0.003	0.003	0.003
35	G	G	G	G	G	T	A	G	G	A	C	C	A	A	T	0.003	-	-	0.003	0.003	0.003
36	G	G	G	G	T	T	A	G	G	G	T	G	A	A	G	0.003	-	-	-	0.003	0.003
	Incorrect Haplotypes																				
1	G	-	G	G	T	T	G	G	G	G	A	G	G	C	T		-	-	-	0.002	0.002
2	G	G	G	G	T	T	G	G	-	A	A	G	A	A	T		-	0.006	-	0.009	0.008
3	G	G	G	G	T	T	G	T	-	G	A	G	A	A	T		-	-	-	-	0.002
4	G	-	G	G	T	T	G	T	G	A	A	G	A	A	T		-	-	0.003	-	-
5	G	-	G	G	T	T	G	T	G	G	A	G	A	A	T		-	-	-	0.003	0.002
6	G	G	G	G	T	T	G	T	G	G	A	G	A	A	T		-	0.003	-	-	-
7	G	G	G	G	T	T	G	T	G	A	A	G	A	A	T		-	-	-	-	0.004
8	A	G	G	G	T	T	A	T	G	A	A	G	A	A	T		-	-	0.003	0.003	0.003
9	G	G	G	G	T	T	A	G	G	G	C	C	A	A	T		0.003	0.003	-	-	-
10	A	G	T	T	G	T	A	G	-	A	A	G	A	A	T		-	-	-	0.002	-
11	A	G	T	T	G	T	A	G	G	G	T	G	A	A	T		-	-	-	0.003	-
12	G	G	T	T	T	T	G	G	-	G	A	G	A	A	T		-	-	0.006	0.004	-
13	G	G	T	T	T	T	G	G	G	A	A	G	A	A	T		-	-	-	0.003	-
14	G	G	G	G	G	T	A	T	-	A	A	G	A	A	T		0.010	-	-	-	-
15	G	G	G	G	G	T	A	T	G	G	A	G	A	A	T		0.006	0.006	0.006	0.008	0.008
16	G	G	G	G	G	T	A	T	G	A	A	G	A	A	G		0.006	0.006	0.006	-	-
	Minor Allele Frequency																				
	0.013	0.036	0.247	0.247	0.399	0.019	0.114	0.367	0.133	0.412	0.065	0.003	0.019	0.019	0.049						

### Accuracy of haplotype inference

The accuracy of computational inferences of haplotype frequencies and assignments to individuals were compared to empirical values for the full set of 15 SNPs in the promoter of GH1. Additionally, analyses were performed on a restricted set of eight SNPs with allele frequencies above 5% (and excluding site -301 which is in complete linkage disequilibrium with -308). The latter analyses were performed to better approximate the characteristics of data sets that are typically collected in genetic epidemiological studies. Although the presence of rare alleles and haplotypes improves the accuracy of haplotype inference, sites with a low frequency minor allele are often ignored due to their reduced usefulness in mapping disease loci and the assumption that such loci will contribute little to population-wide predisposition to disease. Very little difference was observed in the accuracy of haplotype inference between the two data sets.

Assignment of haplotypes to individuals was very accurate by all methods (Table 3). Approximately 90% of individuals were assigned correct haplotypes. However, this number includes individuals whose haplotypes are unambiguous (heterozygotes at 0 or 1 site). Excluding those individuals, the error rate is closer to 13%.

**Table 3:** Accuracy of computational inferences of haplotype structure of the GH1 gene promoter.

		15 Promoter SNPs				Error Rate Based on # of Heterozygous Sites (N)				
			
				Error Rate						
									
Algorithm	MSE	I_H _# correct/# wrong	I_F_	Overall	Ambiguous Individuals	2 (13)	3 (32)	4 (24)	5 (28)	6 (11)
Phase v2, no LD	3.6 × 10^-5^	0.81 27/4	0.91	0.11	0.15	0.08	0.09	0.25	0.11	0.27
Phase v2, with LD	2.2 × 10^-5^	0.81 28/5	0.93	0.10	0.13	0	0.09	0.17	0.14	0.27
Haplotyper 1.0	2.0 × 10^-5^	0.81 28/5	0.93	0.09	0.13	0	0.06	0.13	0.18	0.27
PL-EM 1.0	2.5 × 10^-5^	0.82 31/9	0.92	0.11	0.15	0	0.09	0.13	0.21	0.36
SNPHAP 1.0	2.0 × 10^-5^	0.82 30/7	0.93	0.09	0.13	0	0.09	0.13	0.14	0.27
		8 SNPs with Minor Allele Frequency ≥ 5%								
							
Phase v2, no LD	4.8 × 10^-5^	0.79 19/3	0.92	0.11	0.15					
Phase v2, with LD	4.1 × 10^-5^	0.80 20/4	0.92	0.11	0.15					
Haplotyper 1.0	3.8 × 10^-5^	0.81 19/2	0.93	0.10	0.14					
PL-EM 1.0	2.3 × 10^-5^	0.85 22/4	0.94	0.08	0.11					
SNPHAP 1.0	3.3 × 10^-5^	0.85 22/4	0.93	0.08	0.11					

Estimation of haplotype frequencies was also highly accurate, and there was no meaningful difference in accuracy among the methods as measured by the similarity index, I_F_. As measured by the mean squared error (MSE) the implementation of the program Phase that ignored linkage disequilibrium among sites gave marginally lower accuracy for the full data set and for the data set composed of higher frequency alleles, but the magnitude of the MSE was small for all methods and spanned only about a two-fold difference between the best and worst value. PL-EM successfully identified the largest number of correct haplotypes, but this success rate was accompanied by the burden of the highest number of incorrect haplotypes inferred. Indeed, the aggregate frequency of incorrect haplotypes inferred by PL-EM was about 1% higher than for the other methods. This observation may have practical value for the analysis of unphased genotypic data. PL-EM may be slightly advantageous if the analytical goal of identify the largest number of correct haplotypes is much more important than minimization of the number of incorrect haplotypes inferred, which may be the case in studies of functional genetics. However, if minimization of the number of incorrect low-frequency haplotypes is more important, as will usually be the case in genetic epidemiological studies, PL-EM may not be the optimal method. Unfortunately, none of the methods is clearly superior in minimizing the number of incorrect haplotypes inferred.

Importantly, none of the methods failed to identify haplotypes with frequencies above 1%. Conversely, no incorrect haplotype was assigned a frequency greater than 1%. Indeed, the aggregate frequency of incorrect haplotypes was ≤ 3.7% by all methods. These results are reassuring in two respects. First, it appears unlikely that any of the methods will fail to identify a haplotype that is a major contributor to disease risk within a study population. Second, it also is unlikely that an incorrect haplotype will be implicated as a significant disease risk.

It has been noted previously [17,18] that computational methods tend to over-estimate slightly the frequency of the more common haplotypes. The four most common haplotypes in this data set have an aggregate frequency of 64%. The aggregate frequency inferred for these haplotypes ranged from 63% to 65.9% among the methods. The magnitude of error in the estimation of the frequency of the common haplotypes is very small and indicates that this should not be a significant source of error in studies of population genetics or genetic epidemiology if the present results can be generalized.

### Effect of number of heterozygous sites

The number of possible haplotypes compatible with an individual's unphased genotype is 2^k^, where *k *is the number of heterozygous sites. For this reason, the difficulty of correctly assigning haplotypes to subjects increases dramatically as those subjects become heterozygous at more sites. Therefore, the error rate for assigning haplotypes was evaluated based on the number of sites at which subjects were heterozygous. Up to 3 heterozygous sites, the error rate is below 10%. For 4 heterozygous sites the error rate is about 15% and exceeds 20% only when 6 sites are heterozygous. Phase was an odd exception to this pattern due to an unusually high error rate for four heterozygous sites, despite the lowest error rates for five and six heterozygous sites. On the assumption that an error rate not much larger than 10% is desirable for a genetic study, it appears that computationally assigned haplotypes for subjects heterozygous at more than four SNPs should be viewed with extreme caution. Similarly, there is a current effort to define haplotype blocks in the human genome to facilitate genome-wide scans for disease loci with a minimum number of sites that must be genotyped. If the results for this gene can be generalized, it would appear unwise to define haplotype blocks based on more than 4–5 SNPs.

## Conclusions

All of the implementations of the EM and Bayesian methods of haplotype inference had high accuracy. Therefore, if this data set is representative of other SNP genotyping studies one can have high confidence in the assignment of haplotypes and estimation of haplotype frequencies produced by any one of the programs. Each method identified every haplotype with a frequency greater than 1%. Therefore, it is unlikely that any of the methods would fail to identify a haplotype contributing to disease risk in a significant proportion of a population. Conversely, no incorrect haplotype was assigned a frequency greater than 1%, indicating a low probability of an incorrect haplotype being identified as a significant disease risk factor. Assignment of haplotypes was very accurate for subjects heterozygous for up to three SNPs, and was at least 80% accurate for up to five heterozygous sites. This suggests that haplotype blocks should perhaps be defined based on no more than five sites and that this might be the practical limit at which one can have confidence in the assignment of haplotypes to subjects.

## Methods

### Genetic analyses

The empirically determined set of haplotypes from Horan et al. [23] were kindly provided by Drs. David Cooper and David Millar. To examine the accuracy of computational haplotype inference, five different algorithms (Table 3) were used to infer haplotypes based on the 15 SNPs scored by Horan et al. (2003), and the accuracy of these haplotypes was compared to their empirical determinations. The program HAPLOTYPER [13] takes a Bayesian approach to haplotype inference and a partition-ligation strategy for improving speed and accuracy that divides the data into small segments of consecutive loci during haplotype inference that are later combined. We used the default settings of the htyperv2 program, except that 50 iterations of prediction were requested before the results were reported. Like HAPLOTYPER, Phase 2.0.2 [15] employs a Bayesian approach and partition-ligation. Phase was run both with and without the assumption of decay of linkage disequilibrium (option M) with distance in order to evaluate the effect of this assumption. Phase was run with the default options with these exceptions: five restarting points (-x option), the triallelic site -1 was treated as multiallelic but without the stepwise mutation model (-d option), ten steps through the Markov chain per iteration ("thinning intervals"), and the length of the final run with all loci increased by tenfold (option -X). According to Stephens and Donnelly (2003) HAPLOTYPER and Phase differ primarily in the prior distribution that is used. Phase uses an approximate coalescent that will give greater weight to haplotype resolutions of multilocus genotypes that are most similar to previously resolved haplotypes, while HAPLOTYPER uses a Dirichlet prior that chooses randomly among possible haplotype resolutions if the genotypes can not be made to correspond to previously inferred haplotypes. The program PL-EM [12] combines partition-ligation with the EM algorithm to infer haplotypes. PL-EM was run with these settings: haplotypes with probability of appearance >0.1 reported, 3–4 loci per partition, 154 partial haplotypes passed on in each ligation step, 50 independent runs in each implementation of the EM algorithm. The program SNPHAP [11] by David Clayton also employs the EM algorithm to infer haplotypes, but differs from many implementations by adding one locus at a time and removing from consideration low probability haplotypes after each addition until all loci are added. The default settings for SNPHAP were used. Another popular implementation of the EM algorithm, EM-DeCODER [13], is limited to 100 genotypes and could not be applied to the full set of 154 subjects of Horan et al. (2003).

The full data set of Horan et al. [23] includes six sites with a minor allele frequency below 5%. Sites with allele frequencies this low are often ignored in genetic studies. Therefore, haplotypes were also inferred based upon a restricted set of sites that excluded six sites (-476, -339, -168, +3, +16, and +25) with minor allele frequencies below 5% and excluded site -301 which is in complete linkage disequilibium with site -308. Additionally, the single individual bearing a C allele at sites +1 and +3 was excluded due to the extremely low frequency of that allele. This left eight sites upon which to perform haplotype inference.

Pairwise D' [25], the linkage disequilibium statistic D standardized by its maximum value, was calculated for loci with minor allele frequencies above 5% using the program Arlequin v2.000 [26] based on the empirical haplotypes provided by Drs. Cooper and Millar.

### Measures of accuracy of haplotype inference

The accuracy of haplotype inference was examined by several metrics. The mean squared error (MSE) [18] is defined as



where *p*_*ek *_and *p*_*tk *_are the inferred and empirically determined frequencies for the k*th *haplotype, and *h *is the number of haplotypes. *I*_*F *_and *I*_*H *_were proposed by Excoffier and Slatkin [10]. *I*_*F *_is another measure of how closely the inferred and empirical haplotype frequencies correspond and is given by



where the variables are defined as above. *I*_*F *_ranges from 0 to a maximum value of 1 when the frequencies match perfectly. *I*_*H *_compares the number of haplotypes inferred to the number actually known to occur and ranges from 0 to 1 (complete correspondence between inferred and true). *I*_*H *_is defined as



where *m*_*true *_is the number of haplotypes known to occur, *m*_*est *_is the number of inferred haplotypes with frequency ≥ 1/(2*n*), and *m*_*missed *_is the number of known haplotypes that were not inferred. The error rate [13] is the proportion of subjects whose inferred haplotypes are not completely accurate.
